# Analytical Approximant to a Quadratically Damped Forced Cubic-Quintic Duffing Oscillator

**DOI:** 10.1155/2022/8125305

**Published:** 2022-09-13

**Authors:** Alvaro H. S. Salas

**Affiliations:** Universidad Nacional de Colombia, Fizmako Research Group, Bogota, Colombia

## Abstract

The cubic-quintic Duffing oscillator of a system with strong quadratic damping and forcing is considered. We give elementary approximate analytical solution to this oscillator in terms of exponential and trigonometric functions. We compare the analytical approximant with the Runge–Kutta numerical solution. The approximant allows us to estimate the points at which the solution crosses the horizontal axis.

## 1. Introduction

In this paper, some novel analytical and numerical techniques are introduced for analyzing and solving nonlinear ordinary differential equations (NODEs) that are associated to some strongly nonlinear oscillators such as a quadratically damped cubic-quintic Duffing equation. There are many numerical and analytical approaches that were applied for solving the second-order nonlinear oscillator equations. For instance, both the homotopy perturbation method (HBM) and MTS technique were applied for analyzing a forced Van der Pol (VdP) generalized oscillator to obtain the amplitudes of the forced harmonic and super and subharmonic oscillatory states[1]. Also, Melnikov's method was employed for analyzing a mVdPD equation and deriving analytical criteria for the appearance of horseshoe chaos in chemical oscillations [2]. He et al. [3] used the Poincare–Lindstedt technique (PLT) for solving and analyzing the hybrid Rayleigh–Van der Pol–Duffing equation. Moreover, the homotopy analysis method (HAM) was employed for analyzing the DVdP oscillator [4]. Both methods of differentiable dynamics and Lie symmetry reduction method were devoted for analyzing the DVdP-type oscillator [5]. The principal feature associated with quadratic damping is a discontinuous jump of the damping force in the equation of motion whenever the velocity vanishes such that the frictional force always opposes the motion.

In this paper, we will consider the following quadratically damped and forced cubic-quintic Duffing oscillator:

(1) $$\begin{array}{c} \ddot{x}+\varepsilon \dot{x}\left|\dot{x}\right|+\alpha x+\beta x^{3}+\gamma x^{5}=F\left(t\right),x\left(0\right)=x_{0}\text{ and }x^{\prime }\left(0\right)={\dot{x}}_{0}. \end{array}$$

The quadratically damped oscillator (1) is never critically damped or overdamped. In the absence of damping and forcing, we obtain the cubic-quintic Duffing equation

(2) $$\begin{array}{c} \ddot{x}+\alpha x+\beta x^{3}+\gamma x^{5}=0,x\left(0\right)=x_{0}\text{ and }x^{\prime }\left(0\right)={\dot{x}}_{0}. \end{array}$$

Equation (2) admits exact analytical solution that is expressed in terms of the Jacobian elliptic functions. Other solution methods may be found in [1–8].

## 2. Solution Procedure

### 2.1. First Case: Undamped and Unforced Cubic-Quintic Duffing Equation

Let us consider the i.v.p.

(3) $$\begin{array}{c} \ddot{x}+\alpha x+\beta x^{3}+\gamma x^{5}=0,x\left(0\right)=x_{0}\text{ and }x^{\prime }\left(0\right)={\dot{x}}_{0}. \end{array}$$

Assume the ansatz

(4) $$\begin{array}{c} x\left(t\right)=\frac{v\left(t\right)}{\sqrt{1+\lambda v^{2}\left(t\right)+\mu v^{4}\left(t\right)}}, \end{array}$$

where the function *v*=*v*(*t*) is the solution to some Duffing equation

(5) $$\begin{array}{c} \ddot{v}+pv+qv^{3}=0,v\left(0\right)=v_{0}\text{ and }v^{\prime }\left(0\right)={\dot{v}}_{0}. \end{array}$$

The numbers *v*_0_ and ${\dot{v}}_{0}$ are determined from the initial conditions. Observe that

(6) $$\begin{array}{c} {\dot{v}}_{0}=-\frac{{\dot{x}}_{0}{\left(\lambda v_{0}^{2}+\mu v_{0}^{4}+1\right)}^{3/2}}{\mu v_{0}^{4}-1}. \end{array}$$

From (5), it follows that

(7) $$\begin{array}{c} {\dot{v}}^{2}={\dot{v}}_{0}^{2}+pv_{0}^{2}+\frac{qv_{0}^{4}}{2}-pv^{2}-\frac{qv^{4}}{2}. \end{array}$$

Observe that

(8) $$\begin{array}{c} \frac{1}{2}{\dot{x}}^{2}+\frac{1}{2}\alpha x^{2}+\frac{1}{4}\beta x^{4}+\frac{1}{6}\gamma x^{6}-\frac{\alpha x_{0}^{2}}{2}-\frac{\beta x_{0}^{4}}{4}-\frac{1}{6}\gamma x_{0}^{6}-\frac{{\dot{x}}_{0}^{2}}{2}=0. \end{array}$$

We have

(9) $$\begin{array}{c} 0=\frac{1}{2}{\dot{x}}^{2}+\frac{1}{2}\alpha x^{2}+\frac{1}{4}\beta x^{4}+\frac{1}{6}\gamma x^{6}-\frac{\alpha x_{0}^{2}}{2}-\frac{\beta x_{0}^{4}}{4}-\frac{1}{6}\gamma x_{0}^{6}-\frac{{\dot{x}}_{0}^{2}}{2}=\frac{1}{12\left(1+\lambda v^{2}+\mu v^{4}\right)}\sum_{j=0}^{6}S_{j}v^{2j}, \end{array}$$

where

(10) $$\begin{array}{c} S_{0}=6pv_{0}^{2}+3qv_{0}^{4}+6{\dot{v}}_{0}^{2}-6\alpha x_{0}^{2}-3\beta x_{0}^{4}-2\gamma x_{0}^{6}-6{\dot{x}}_{0}^{2},\\ S_{1}=-3\left(-2\alpha +2p+6\alpha \lambda x_{0}^{2}+3\beta \lambda x_{0}^{4}+2\gamma \lambda x_{0}^{6}+6\lambda {\dot{x}}_{0}^{2}\right),\\ S_{2}=-3\left(-4\alpha \lambda -\beta +4\mu pv_{0}^{2}+2\mu qv_{0}^{4}+q+4\mu {\dot{v}}_{0}^{2}+6\alpha \lambda^{2}x_{0}^{2}+6\alpha \mu x_{0}^{2}+3\beta \lambda^{2}x_{0}^{4}+3\beta \mu x_{0}^{4}+2\gamma \lambda^{2}x_{0}^{6}+2\gamma \mu x_{0}^{6}+6\lambda^{2}{\dot{x}}_{0}^{2}+6\mu {\dot{x}}_{0}^{2}\right),\\ S_{3}=6\alpha \lambda^{2}+12\alpha \mu +3\beta \lambda +2\gamma +12\mu p-6\alpha \lambda^{3}x_{0}^{2}-36\alpha \lambda \mu x_{0}^{2}-3\beta \lambda^{3}x_{0}^{4}-18\beta \lambda \mu x_{0}^{4}-2\gamma \lambda^{3}x_{0}^{6}-12\gamma \lambda \mu x_{0}^{6}-6\lambda^{3}{\dot{x}}_{0}^{2}-36\lambda \mu {\dot{x}}_{0}^{2},\\ S_{4}=3\mu \left(4\alpha \lambda +\beta +2\mu pv_{0}^{2}+\mu qv_{0}^{4}+2q+2\mu {\dot{v}}_{0}^{2}-6\alpha \lambda^{2}x_{0}^{2}-6\alpha \mu x_{0}^{2}-3\beta \lambda^{2}x_{0}^{4}-3\beta \mu x_{0}^{4}-2\gamma \lambda^{2}x_{0}^{6}-2\gamma \mu x_{0}^{6}-6\lambda^{2}{\dot{x}}_{0}^{2}-6\mu {\dot{x}}_{0}^{2}\right),\\ S_{5}=-3\mu^{2}\left(-2\alpha +2p+6\alpha \lambda x_{0}^{2}+3\beta \lambda x_{0}^{4}+2\gamma \lambda x_{0}^{6}+6\lambda {\dot{x}}_{0}^{2}\right),\\ S_{6}=-\mu^{2}\left(3q+6\alpha \mu x_{0}^{2}+3\beta \mu x_{0}^{4}+2\gamma \mu x_{0}^{6}+6\mu {\dot{x}}_{0}^{2}\right). \end{array}$$

Eliminating *p*, *q*, *μ*, *v*_0_, and ${\dot{v}}_{0}$ from the system *S*_0_=*S*_1_=*S*_2_=*S*_3_=*S*_4_=*S*_5_=*S*_6_=0 gives

(11) $$\begin{array}{c} \left(\left(6\alpha x_{0}^{2}+3\beta x_{0}^{4}+2\gamma x_{0}^{6}+6{\dot{x}}_{0}^{2}\right)\lambda^{3}-6\alpha \lambda^{2}-3\beta \lambda -2\gamma \right)\left(\begin{array}{c} 4{\left(6\alpha x_{0}^{2}+3\beta x_{0}^{4}+2\gamma x_{0}^{6}+6{\dot{x}}_{0}^{2}\right)}^{2}\lambda^{3}-24\alpha \left(6\alpha x_{0}^{2}+3\beta x_{0}^{4}+2\gamma x_{0}^{6}+6{\dot{x}}_{0}^{2}\right)\lambda^{2}\\ +3\left(12\alpha^{2}-6\alpha \beta x_{0}^{2}-3\beta^{2}x_{0}^{4}-2\beta \gamma x_{0}^{6}-6\beta {\dot{x}}_{0}^{2}\right)\lambda +9\alpha \beta +6\alpha \gamma x_{0}^{2}+3\beta \gamma x_{0}^{4}+2\gamma^{2}x_{0}^{6}+6\gamma {\dot{x}}_{0}^{2} \end{array}\right)=0. \end{array}$$

The values for *p*, *q*, and *μ* are

(12) $$\begin{array}{c} p=\frac{1}{2}\left(2\alpha -6\alpha \lambda x_{0}^{2}-3\beta \lambda x_{0}^{4}-2\gamma \lambda x_{0}^{6}-6\lambda {\dot{x}}_{0}^{2}\right),\\ q=\frac{1}{3}\left(12\alpha \lambda +3\beta -18\alpha \lambda^{2}x_{0}^{2}-30\alpha \mu x_{0}^{2}-9\beta \lambda^{2}x_{0}^{4}-15\beta \mu x_{0}^{4}-6\gamma \lambda^{2}x_{0}^{6}-10\gamma \mu x_{0}^{6}-18\lambda^{2}{\dot{x}}_{0}^{2}-30\mu {\dot{x}}_{0}^{2}\right),\\ \mu =\frac{6\alpha \lambda^{2}+3\beta \lambda +2\gamma -6\alpha \lambda^{3}x_{0}^{2}-3\beta \lambda^{3}x_{0}^{4}-2\gamma \lambda^{3}x_{0}^{6}-6\lambda^{3}{\dot{x}}_{0}^{2}}{12\left(-2\alpha +6\alpha \lambda x_{0}^{2}+3\beta \lambda x_{0}^{4}+2\gamma \lambda x_{0}^{6}+6\lambda {\dot{x}}_{0}^{2}\right)}. \end{array}$$

The value of *v*_0_ is found from the condition *x*(0)=*x*_0_:

(13) $$\begin{array}{c} v_{0}=\pm \sqrt{\frac{1-\lambda x_{0}^{2}\pm \sqrt{{\left(\lambda x_{0}^{2}-1\right)}^{2}-4\mu x_{0}^{4}}}{2\mu x_{0}^{2}}}. \end{array}$$

On the other hand, the solution to i.v.p. (5) is given by

(14) $$\begin{array}{c} v\left(t\right)=\text{ccn}\left(\sqrt{qc^{2}+pt}+cn^{-1}\left(\frac{v_{0}}{c},\frac{c^{2}q}{2\left(qc^{2}+p\right)}\right),\frac{c^{2}q}{2\left(qc^{2}+p\right)}\right), \end{array}$$

where

(15) $$\begin{array}{c} c=\pm \sqrt{\frac{-p\pm \sqrt{{\left(p+qv_{0}^{2}\right)}^{2}+2q{\dot{v}}_{0}^{2}}}{q}.} \end{array}$$

The number

(16) $$\begin{array}{c} \Delta ={\left(p+qv_{0}^{2}\right)}^{2}+2q{\dot{v}}_{0}^{2}, \end{array}$$

is called the discriminant to Duffing equation (5). In the case when Δ > 0 , this solution may also be written in the form

(17) $$\begin{array}{c} v\left(t\right)=\frac{v_{0}cn\left(t\sqrt{\omega }|m\right)+\left({\dot{v}}_{0}/\sqrt{\omega }\right)dn\left(t\sqrt{\omega }|m\right)sn\left(t\sqrt{\omega }|m\right)}{1+\left(\left(p+qv_{0}^{2}-\omega \right)/2\omega \right)sn{\left(t\sqrt{\omega }|m\right)}^{2}}, \end{array}$$

where

(18) $$\begin{array}{c} \omega =-\frac{p}{2m-1},\;\\ m=\frac{1}{2}\left(1\pm \frac{p}{\sqrt{{\left(p+qv_{0}^{2}\right)}^{2}+2q{\dot{v}}_{0}^{2}}}\right). \end{array}$$

Suppose that Δ < 0. Define

(19) $$\begin{array}{c} \delta =2pv_{0}^{2}+qv_{0}^{4}+2{\dot{v}}_{0}^{2}. \end{array}$$

Since Δ < 0, necessarily *q* < 0. From the equality

(20) $$\begin{array}{c} \delta =\frac{p^{2}-\Delta }{-q}, \end{array}$$

it is evident that *δ* > 0.Then, the solution to i.v.p. (5) reads

(21) $$\begin{array}{c} v\left(t\right)=\sqrt[{4}]{\frac{\delta }{-q}}-\frac{2\sqrt[{4}]{\delta /-q}}{\left(1+u_{0}cn\left(\sqrt{\bar{\omega }}x|\bar{m}\right)+\left({\dot{u}}_{0}/\sqrt{\omega }\right)sn\left(\sqrt{\bar{\omega }}x|\bar{m}\right)dn\left(\sqrt{\bar{\omega }}x|\bar{m}\right)\right)/\left(1+\bar{b}sn{\left(\sqrt{\bar{\omega }}x|\bar{m}\right)}^{2}\right)}, \end{array}$$

where

(22) $$\begin{array}{c} \bar{\omega }=\sqrt{\bar{\Delta }},\\ \bar{m}=\frac{1}{2}\pm \frac{\bar{\alpha }}{2\sqrt{\bar{\Delta }}},\\ \bar{b}=\frac{\bar{\alpha }+\bar{\beta }u_{0}^{2}}{2\sqrt{\bar{\Delta }}}-\frac{1}{2},\\ u_{0}=\frac{A+v_{0}}{A-v_{0}}\;and\;{\dot{u}}_{0}=\frac{2A\;{\dot{v}}_{0}^{2}}{{\left(A-v_{0}\right)}^{2}},\\ \bar{\Delta }={\left(\bar{\alpha }+u_{0}^{2}\bar{\beta }\right)}^{2}+2\bar{\beta }\;{\dot{u}}_{0}^{2},\\ \bar{\alpha }=\frac{1}{2}\left(3A^{2}q-p\right),\\ \bar{\beta }=\frac{1}{2}\left(A^{2}q+p\right),\\ A=\sqrt[{4}]{\frac{\delta }{-q}},\\ \delta =2pv_{0}^{2}+qv_{0}^{4}+2{\dot{v}}_{0}^{2}. \end{array}$$

Observe that

(23) $$\begin{array}{c} \bar{\Delta }={\left(\bar{\alpha }+u_{0}^{2}\bar{\beta }\right)}^{2}+2\bar{\beta }\;{\dot{u}}_{0}^{2}=2\left(\sqrt{p^{2}\left(-q\right)\delta }+\left(-q\right)\delta \right)>0. \end{array}$$

**Example 1 .**

Let us consider the i.v.p.

(24) $$\begin{array}{c} \ddot{x}-1.25819x-2.40487x^{3}+0.299493x^{5}=0,x\left(0\right)=-0.261431\text{ and }x^{\prime }\left(0\right)=0.770472. \end{array}$$

The exact solution is given by

(25) $$\begin{array}{c} x_{\text{exact}}\left(t\right)=\frac{v\left(t\right)}{\sqrt{1-1.2914v^{2}\left(t\right)+0.231351v^{4}\left(t\right)}}, \end{array}$$

where

(26) $$\begin{array}{c} v\left(t\right)=\frac{-0.250491cn\left(0.916322t|0.0909919\right)+0.738957\;dn\left(0.916322t|0.0909919\right)sn\left(0.916322t|0.0909919\right)}{1-0.0823122sn^{2}\left(0.916322t|0.0909919\right)}. \end{array}$$

See Figure 1.

### 2.2. Solution by Means of He's Frequency Method

Let

(27) $$\begin{array}{c} f\left(x\right)=\alpha x+\beta x^{3}+\gamma x^{5}. \end{array}$$

He's method assumes the solution in the ansatz form

(28) $$\begin{array}{c} x\left(t\right)=A\;\cos\left(\sqrt{\omega }t+\;\cos^{-1}\left(\frac{x_{0}}{A}\right)\right),\;A\neq 0. \end{array}$$

The frequency is evaluated by means of the formula

(29) $$\begin{array}{c} \omega =\frac{f\left(x\right)}{x}\;\text{at}\;x=\frac{\sqrt{3}}{2}A. \end{array}$$

Then,

(30) $$\begin{array}{c} \omega =\omega_{He}=\alpha +\frac{3\beta }{4}A^{2}+\frac{9\gamma }{16}A^{4}. \end{array}$$

The number *A* is found from the initial condition $x^{\prime }\left(0\right)={\dot{x}}_{0}$ so that

(31) $$\begin{array}{c} A=\pm \sqrt{\frac{{\dot{x}}_{0}^{2}}{\alpha +\left(9A^{4}\;\gamma /16\right)+\left(3A^{2}\beta /4\right)}+x_{0}^{2}}. \end{array}$$

### 2.3. Solution by Means of a Simple Trigonometric Ansatz

As in He's approach, we assume the solution in ansatz form (28) so that

(32) $$\begin{array}{c} \ddot{x}+\alpha x+\beta x^{3}+\gamma x^{5}=\frac{1}{16}A^{5}\gamma \;\cos\left(5\theta \right)=+\frac{1}{16}\cos\left(3\theta \right)\left(5A^{5}\gamma +4A^{3}\beta \right)+\frac{1}{8}A\;\cos\left(\theta \right)\left(8\alpha +5A^{4}\gamma +6A^{2}\beta -8\omega \right). \end{array}$$

We choose the frequency *ω* so that

(33) $$\begin{array}{c} 8\alpha +5A^{4}\gamma +6A^{2}\beta -8\omega =0. \end{array}$$

Then,

(34) $$\begin{array}{c} \omega =\omega_{\text{trigo}}=\alpha +\frac{3\beta }{4}A^{2}+\frac{10\gamma }{16}A^{4}. \end{array}$$

This last formula looks like He's formula (30). The difference is *ω*_trigo_ − *ω*_He_=(*γ*/16)*A*^4^. This suggests to consider the following *κ*-parameter solution:

(35) $$\begin{array}{c} x\left(\kappa ,t\right)=x\left(t\right)=A\;\cos\left(\sqrt{\alpha +\frac{3\beta }{4}A^{2}+\frac{9\gamma }{16}\kappa A^{4}t}+\;\cos^{-1}\left(\frac{x_{0}}{A}\right)\right), \end{array}$$

where the number *A* is a solution to the sextic

(36) $$\begin{array}{c} \frac{1}{16}\gamma \lambda A^{6}+\left(\frac{3\beta }{4}-\frac{1}{16}\gamma \lambda x_{0}^{2}\right)A^{4}+\left(\alpha -\frac{3\beta x_{0}^{2}}{4}\right)A^{2}-\alpha x_{0}^{2}-{\dot{x}}_{0}^{2}=0. \end{array}$$

The number *λ* is chosen in order to get as small residual error as possible.

### 2.4. Solution by Means of an Improved Trigonometric Ansatz

#### 2.4.1. First Improved Ansatz

Let us consider the i.v.p.

(37) $$\begin{array}{c} \ddot{x}+\alpha x+\beta x^{3}+\gamma x^{5}=0,x\left(0\right)=A\text{ and }x^{\prime }\left(0\right)=0. \end{array}$$

Assume the ansatz

(38) $$\begin{array}{c} x\left(t\right)=A\frac{\sqrt{1+\lambda +\mu }\cos\left(\sqrt{\omega t}\right)}{\sqrt{1+\lambda \;\cos^{2}\left(\sqrt{\omega t}\right)+\mu \;\cos^{4}\left(\sqrt{\omega t}\right)}}. \end{array}$$

Let

(39) $$\begin{array}{c} R\left(t\right)=x^{\prime \prime }\left(t\right)+\alpha x\left(t\right)+\beta x^{3}\left(t\right)+\gamma x^{5}\left(t\right). \end{array}$$

The numbers *λ*, *μ*, and *ω* are found from the conditions

(40) $$\begin{array}{c} R\left(0\right)=R^{\prime \prime }\left(0\right)=R^{\prime \prime \prime \prime }\left(0\right)=0,\\ \lambda =-\frac{2\left(4\alpha^{2}-\alpha \omega +2A^{8}\gamma^{2}+5A^{6}\;\beta \gamma +6\alpha A^{4}\gamma +3A^{4}\beta^{2}-A^{4}\gamma \omega +7\alpha A^{2}\beta -A^{2}\beta \omega -3\omega^{2}\right)}{\left(\alpha +A^{4}\gamma +A^{2}\beta \right)\left(4\alpha +2A^{4}\gamma +3A^{2}\beta +2\omega \right)},\\ \mu =\frac{4\alpha +2A^{4}\gamma +3A^{2}\beta -4\omega }{4\alpha +2A^{4}\gamma +3A^{2}\beta +2\omega }. \end{array}$$

The frequency *ω* is found from the quadratic equation

(41) $$\begin{array}{c} 34\alpha^{2}+A^{4}\left(2A^{2}\gamma +3\beta \right)\left(5A^{2}\gamma +6\beta \right)+\alpha \left(40A^{4}\gamma +51A^{2}\beta \right)-5\left(4\alpha +2A^{4}\gamma +3A^{2}\beta \right)\omega -14\omega^{2}=0. \end{array}$$

#### 2.4.2. Second Improved Ansatz

Let us consider the i.v.p.

(42) $$\begin{array}{c} \ddot{x}+\alpha x+\beta x^{3}+\gamma x^{5}=0\text{,}x\left(0\right)=A\text{ and }x^{\prime }\left(0\right)=0. \end{array}$$

Assume the ansatz

(43) $$\begin{array}{c} x\left(t\right)=A\frac{\sqrt{1+\lambda +\mu +\upsilon }\cos\left(\sqrt{\omega t}\right)}{\sqrt{1+\lambda \;\cos^{2}\left(\sqrt{\omega t}\right)+\mu \;\cos^{4}\left(\sqrt{\omega t}\right)+\upsilon \;\cos^{6}\left(\sqrt{\omega t}\right)}}. \end{array}$$

The numbers *λ*, *μ*, *υ*, and *ω* are found from the conditions

(44) $$\begin{array}{c} R\left(0\right)=R^{\prime \prime }\left(0\right)=R^{\left(4\right)}\left(0\right)=R^{\left(6\right)}\left(0\right)=0,\\ \lambda =-\frac{3\left(\begin{array}{c} 34\alpha^{3}+20\alpha^{2}\omega -24\alpha \omega^{2}+10A^{12}\gamma^{3}+37A^{10}\beta \gamma^{2}+\\ 50\alpha A^{8}\gamma^{2}+45A^{8}\beta^{2}\gamma +10A^{8}\gamma^{2}\omega +118\alpha A^{6}\beta \gamma +\\ 18A^{6}\beta^{3}+25A^{6}\beta \gamma \omega +74\alpha^{2}A^{4}\gamma +69\alpha A^{4}\beta^{2}+30\alpha A^{4}\gamma \omega +\\ 15A^{4}\beta^{2}\omega -24A^{4}\gamma \omega^{2}+85\alpha^{2}A^{2}\beta +35\alpha A^{2}\beta \omega -24A^{2}\beta \omega^{2}-30\omega^{3} \end{array}\right)}{\left(\alpha +A^{4}\gamma +A^{2}\beta \right)\left(\begin{array}{c} 34\alpha^{2}+40\alpha \omega +10A^{8}\gamma^{2}+27A^{6}\beta \gamma +\\ 40\alpha A^{4}\gamma +18A^{4}\beta^{2}+20A^{4}\gamma \omega +51\alpha A^{2}\beta +30A^{2}\beta \omega +16\omega^{2} \end{array}\right)},\\ \mu =\frac{3\left(34\alpha^{2}+10A^{8}\gamma^{2}+27A^{6}\beta \gamma +40\alpha A^{4}\gamma +18A^{4}\beta^{2}+51\alpha A^{2}\beta -34\omega^{2}\right)}{34\alpha^{2}+40\alpha \omega +10A^{8}\gamma^{2}+27A^{6}\beta \gamma +40\alpha A^{4}\gamma +18A^{4}\beta^{2}+20A^{4}\gamma \omega +51\alpha A^{2}\beta +30A^{2}\beta \omega +16\omega^{2}},\\ \upsilon =-\frac{34\alpha^{2}-20\alpha \omega +10A^{8}\gamma^{2}+27A^{6}\beta \gamma +40\alpha A^{4}\gamma +18A^{4}\beta^{2}-10A^{4}\gamma \omega +51\alpha A^{2}\beta -15A^{2}\beta \omega -14\omega^{2}}{34\alpha^{2}+40\alpha \omega +10A^{8}\gamma^{2}+27A^{6}\beta \gamma +40\alpha A^{4}\gamma +18A^{4}\beta^{2}+20A^{4}\gamma \omega +51\alpha A^{2}\beta +30A^{2}\beta \omega +16\omega^{2}}. \end{array}$$

The frequency *ω* is found from the cubic equation

(45) $$\begin{array}{c} -\left(4\alpha +3A^{2}\beta +2A^{4}\gamma \right)\left(124\alpha^{2}+186A^{2}\alpha \beta +63A^{4}\beta^{2}+160A^{4}\alpha \gamma +102A^{6}\beta \gamma +40A^{8}\gamma^{2}\right)+84\left(4\alpha +3A^{2}\beta +2A^{4}\gamma \right)\omega +160\omega^{3}=0. \end{array}$$

### 2.5. Homotopy Method

Consider the homotopy

(46) $$\begin{array}{c} H\left(x,p\right)=\ddot{x}+\alpha x+p\left(\beta x^{3}+\gamma x^{5}\right), \end{array}$$

and assume the solution in then ansatz form

(47) $$\begin{array}{c} x\left(t\right)=y_{0}\left(\omega t\right)+py_{1}\left(\omega t\right)+p^{2}y_{2}\left(\omega t\right)+p^{3}y_{3}\left(\omega t\right)+p^{4}y_{4}\left(\omega t\right)+p^{5}y_{5}\left(\omega t\right)+\cdots , \end{array}$$

where $\omega =\sqrt{\alpha +p\omega_{1}+p^{2}\omega_{2}+p^{3}\omega_{2}+p^{4}\omega_{2}+p^{5}\omega_{2}+\cdots }$.

Plugging the expression for *x*(*t*) into *H*(*x*, *p*) and equating the coefficients of *p*^*j*^(*j*=0,1,2,3,…) will give an ode system. Solving this system so that no secular terms appear will give the following expressions: *y*.

### 2.6. Second Case: Quadratically Damped and Unforced Oscillator

Our aim is to give *approximate* analytical solution to i.v.p. (1). Define the residual function *R*=*R*(*t*) as follows:

(48) $$\begin{array}{c} R\left(t\right)=\ddot{x}+\varepsilon \dot{x}\left|\dot{x}\right|+\alpha x+\beta x^{3}+\gamma x^{5}=\ddot{x}\pm \varepsilon {\dot{x}}^{2}+\alpha x+\beta x^{3}+\gamma x^{5}\text{.} \end{array}$$

#### 2.6.1. First Approach

Assume the ansatz

(49) $$\begin{array}{c} x\left(t\right)=c_{0}e^{-\rho t}\cos\left(f\left(t\right)+\;\cos^{-1}\left(\frac{x_{0}}{c_{0}}\right)\right). \end{array}$$

Let *θ*=*f*(*t*)+  cos^−1^(*x*_0_/*c*_0_). We have

(50) $$\begin{array}{c} R\left(t\right)=c_{0}\left(8e^{4t\rho }\;\alpha +8e^{4t\rho }\rho^{2}+6e^{2t\rho }\beta c_{0}^{2}+5\gamma c_{0}^{4}-8e^{4t\rho }f^{\prime }{\left(t\right)}^{2}\right)\cos\left(\theta \right)\\ +\frac{1}{16}e^{-5t\rho }\gamma \;\cos\left(5\theta \right)f^{\prime }\left(t\right)c_{0}^{5}+\frac{1}{16}e^{-5t\rho }c_{0}^{3}\left(4e^{2t\rho }\beta +5\gamma c_{0}^{2}\right)\cos\left(3\theta \right)\\ +e^{-2t\rho }\varepsilon \rho c_{0}^{2}f^{\prime }\left(t\right)\sin\left(2\theta \right)+\frac{1}{2}e^{-2t\rho }\varepsilon c_{0}^{2}\left(\rho^{2}-f^{\prime }{\left(t\right)}^{2}\right)\cos\left(2\theta \right)\\ +\frac{1}{2}e^{-2t\rho }\varepsilon c_{0}^{2}\left(\rho^{2}+f^{\prime }{\left(t\right)}^{2}\right)+\frac{1}{8}e^{-5t\rho }+e^{-t\rho }c_{0}\left(2\rho f^{\prime }\left(t\right)-f^{\prime \prime }\left(t\right)\right)\sin\left(\theta \right). \end{array}$$

We will choose the function *f*=*f*(*t*) so that

(51) $$\begin{array}{c} 8e^{4t\rho }\alpha +8e^{4t\rho }\rho^{2}-8e^{4t\rho }f^{\prime }{\left(t\right)}^{2}+6e^{2t\rho }\beta c_{0}^{2}+5\gamma c_{0}^{4}=0,\text{ and }f\left(0\right)=0\text{ and }f^{\prime }\left(t\right)>0. \end{array}$$

Then,

(52) $$\begin{array}{c} f\left(t\right)=\frac{1}{2\sqrt{2}}\int_{0}^{t}\sqrt{8\alpha +8\rho^{2}+6e^{-2\rho \tau }\beta c_{0}^{2}+5e^{-4\rho \tau }\gamma c_{0}^{4}}d\tau =F\left(t\right)-F\left(0\right), \end{array}$$

where

(53) $$\begin{array}{c} F\left(t\right)=\frac{\sqrt{8\left(\alpha +\rho^{2}\right)+6\beta c_{0}^{2}e^{-2\rho t}+5\gamma c_{0}^{4}e^{-4\rho t}}}{40\sqrt{\gamma }\rho \sqrt{5\gamma c_{0}^{4}+6\beta c_{0}^{2}e^{2\rho t}+8\left(\alpha +\rho^{2}\right)e^{4\rho t}}},\\ \left(\begin{array}{c} -5\sqrt{2}\sqrt{\gamma }\sqrt{5\gamma c_{0}^{4}+6\beta c_{0}^{2}e^{2\rho t}+8\left(\alpha +\rho^{2}\right)e^{4\rho t}}+20\sqrt{\gamma }\sqrt{\alpha +\rho^{2}}e^{2\rho t}\\ {\text{tanh}}^{-1}\left(\frac{3\beta c_{0}^{2}+8\left(\alpha +\rho^{2}\right)e^{2\rho t}}{2\sqrt{2}\sqrt{\alpha +\rho^{2}}\sqrt{5\gamma c_{0}^{4}+6\beta c_{0}^{2}e^{2\rho t}+8\left(\alpha +\rho^{2}\right)e^{4\rho t}}}\right)-3\sqrt{10}\beta e^{2\rho t}{\text{tanh}}^{-1}\left(\frac{5\gamma c_{0}^{2}+3\beta e^{2\rho t}}{\sqrt{5}\sqrt{\gamma }\sqrt{5\gamma c_{0}^{4}+6\beta c_{0}^{2}e^{2\rho t}+8\left(\alpha +\rho^{2}\right)e^{4\rho t}}}\right) \end{array}\right). \end{array}$$

The value of *c*_0_ is found from the initial condition $x^{\prime }\left(0\right)={\dot{x}}_{0}$, and it is a solution to the sextic

(54) $$\begin{array}{c} -8\left(\alpha x_{0}^{2}+2\rho^{2}x_{0}^{2}+2\rho {\dot{x}}_{0}x_{0}+{\dot{x}}_{0}^{2}\right)+2\left(4\alpha +4\rho^{2}-3\beta x_{0}^{2}\right)z^{2}+\left(6\beta -5\gamma x_{0}^{2}\right)z^{4}+5\gamma z^{6}=0. \end{array}$$

The number *ρ* is a free parameter that is chosen in order to minimize the residual error. In particular, when *ε*⟶0, we obtain approximate trigonometric solution to the undamped cubic-quintic Duffing equation

(55) $$\begin{array}{c} \ddot{x}+\alpha x+\beta x^{3}+\gamma x^{5}=0,x\left(0\right)=x_{0}\text{ and }x^{\prime }\left(0\right)={\dot{x}}_{0}. \end{array}$$

**Example 2 .**

Let us consider the i.v.p.

(56) $$\begin{array}{c} \ddot{x}+0.25\left|\dot{x}\right|\dot{x}+x+5x^{3}+10x^{5}=0,x\left(0\right)=0\text{ and }x^{\prime }\left(0\right)=0.1. \end{array}$$

**Figure 2:**
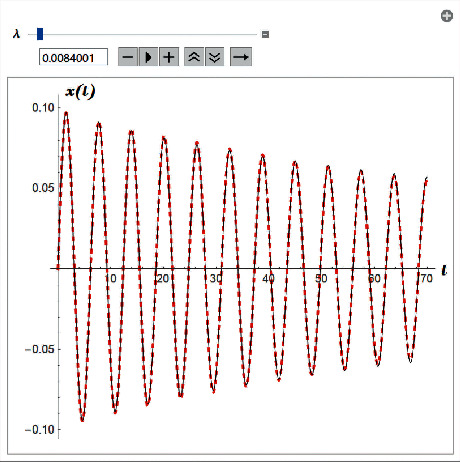
Comparison between analytical approximant (57) and the Runge–Kutta numerical solution for i.v.p. (56).

The approximate analytical solution for *ρ*=0.0084 is

(57) $$\begin{array}{c} u_{\text{approx}}\left(t\right)=x\left(t\right)=-0.0982083e^{-0.0084t}\cos\left(f\left(t\right)+\frac{\pi }{2}\right), \end{array}$$

where the function *f*(*t*) is given by (52) with *ε*=0.25 , *α*=1, *β*=5, *γ*=10, *c*_0_=−0.0983, and *ρ*=0.0084 (see Figure 2).

The obtained results may be applied to solve the pendulum equation with quadratic damping

(58) $$\begin{array}{c} \overset{..}{\theta }+\varepsilon \dot{\theta }\left|\dot{\theta }\right|+\omega^{2}\sin\;\theta =0\text{,}\theta \left(0\right)=\theta_{0}\text{ and }\theta^{\prime }\left(0\right)={\dot{\theta }}_{0}. \end{array}$$

Indeed, we may use the approximation

(59) $$\begin{array}{c} \sin\;\theta \approx \theta -\frac{\theta^{3}}{6}+\frac{\theta^{5}}{131}\text{ for}\left|x\right|\leq \frac{\pi }{2}, \end{array}$$

and then we replace i.v.p. (58) with the i.v.p.

(60) $$\begin{array}{c} \overset{..}{\theta }+\varepsilon \dot{\theta }\left|\dot{\theta }\right|+\omega^{2}\left(\theta -\frac{\theta^{3}}{6}+\frac{\theta^{5}}{131}\right)=0,\theta \left(0\right)=\theta_{0}\text{ and }\theta^{\prime }\left(0\right)={\dot{\theta }}_{0}. \end{array}$$

**Example 3 .**

Let us consider the i.v.p.

(61) $$\begin{array}{c} \overset{..}{\theta }+0.2\dot{\theta }\left|\dot{\theta }\right|+\sin\;\theta =0,\theta =\left(0\right)=20^{{}^{\circ}}\text{ and }\theta^{\prime }\left(0\right)=0.1. \end{array}$$

**Figure 3:**
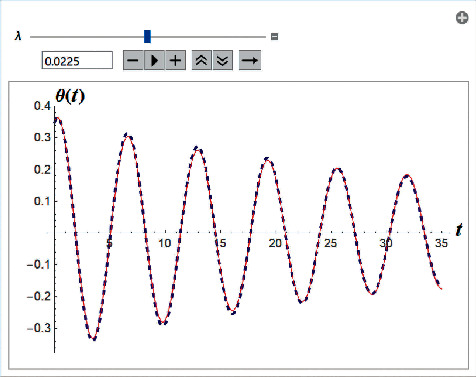
Comparison between the analytical approximant and the Runge–Kutta numerical solution for i.v.p. (61) with *ρ*=0.0225 and *c*_0_=−0.365609.

The optimal value for *ρ* is *ρ* = 0.0225. The value of *c*_0_ is *c*_0_ = −0.365609 (see Figure3).

#### 2.6.2. Second Approach

Let us consider the i.v.p.

(62) $$\begin{array}{c} \ddot{x}+\varepsilon \dot{x}\left|\dot{x}\right|+\alpha x+\beta x^{3}+\gamma x^{5}=0,x\left(0\right)=x_{0}\text{ and }x^{\prime }\left(0\right)={\dot{x}}_{0}. \end{array}$$

Assume the ansatz

(63) $$\begin{array}{c} x\left(t\right)=x_{\rho ,\kappa }\left(t\right)=e^{-\rho t}y\left(\tau \right),\;\tau =\tau \left(t\right)=\frac{1-\exp\left(-\varepsilon \kappa t\right)}{\varepsilon \kappa }, \end{array}$$

where the function *y*=*y*(*τ*) is the exact solution to the i.v.p.

(64) $$\begin{array}{c} \ddot{y}\left(\tau \right)+\alpha y\left(\tau \right)+\beta y^{3}\left(\tau \right)+\gamma y^{5}\left(\tau \right)=0,y\left(0\right)=x_{0}\text{ and }x^{\prime }\left(0\right)={\dot{x}}_{0}+\rho x_{0}. \end{array}$$

The numbers *ρ* and *κ* are free parameters that are chosen in order to minimize the residual error

(65) $$\begin{array}{c} R\left(t\right)=\ddot{x}\left(t\right)+\varepsilon \dot{x}\left(t\right)\left|\dot{x}\left(t\right)\right|+\alpha x\left(t\right)+\beta x^{3}\left(t\right)+\gamma x^{5}\left(t\right). \end{array}$$

Observe that when *ε*⟶0, ((1 − exp(−*εκt*))/*εκ*)⟶*t* and then we obtain the exact solution to the undamped and unforced cubic-quintic oscillator (3). So, we expect accurate approximate analytical solution for small *ε*. This approach is more accurate, but here the solution involves elliptic functions and the solution is not elementary.

#### 2.6.3. Third Approach

Let us consider the i.v.p.

(66) $$\begin{array}{c} \ddot{x}+\varepsilon \dot{x}\left|\dot{x}\right|+\alpha x+\beta x^{3}+\gamma x^{5}=0,x\left(0\right)=x_{0}\text{ and }x^{\prime }\left(0\right)={\dot{x}}_{0}. \end{array}$$

Assume the ansatz

(67) $$\begin{array}{c} x\left(t\right)=x_{\rho ,\lambda }\left(t\right)=ce^{-\rho t}\frac{\cos\left(f\left(t\right)+d\right)}{\sqrt{1+\lambda \;\cos^{2}\left(f\left(t\right)+d\right)}}. \end{array}$$

Proceeding in a similar way as in the first approach, we may choose

(68) $$\begin{array}{c} f\left(t\right)=\int_{0}^{t}\sqrt{\frac{2\left(\lambda +2\right)\left(\alpha +\rho^{2}\right)+\beta c^{2}e^{-2\rho \tau }}{10\lambda +4}}d\tau \;. \end{array}$$

Here we have two free parameters *ρ* and *λ* that are chosen in order to get as less residual error as possible. The numbers *c* and *d* are determined from the initial conditions as follows:

(69) $$\begin{array}{c} d=\pm \;\cos^{-1}\left(\pm \frac{x_{0}}{\sqrt{c^{2}-\lambda x_{0}^{2}}}\right). \end{array}$$

The number *c* is a solution to the octic

(70) $$\begin{array}{c} -2\lambda^{2}\left(\lambda +1\right)\left(\lambda +2\right)x_{0}^{6}\left(\alpha +\rho^{2}\right)+\lambda x_{0}^{4}\left(6\alpha \lambda^{2}+16\alpha \lambda +8\alpha +6\lambda^{2}\rho^{2}+16\lambda \rho^{2}+8\rho^{2}-\beta \lambda^{2}x_{0}^{2}-\beta \lambda x_{0}^{2}\right)c^{2}\\ +\left(\begin{array}{c} -6\alpha \lambda^{2}x_{0}^{2}-14\alpha \lambda x_{0}^{2}-4\alpha x_{0}^{2}+3\beta \lambda^{2}x_{0}^{4}+2\beta \lambda x_{0}^{4}-\\ 6\lambda^{2}\rho^{2}x_{0}^{2}-24\lambda \rho^{2}x_{0}^{2}-20\lambda \rho {\dot{x}}_{0}x_{0}-10\lambda {\dot{x}}_{0}^{2}-8\rho^{2}x_{0}^{2}-8\rho {\dot{x}}_{0}x_{0}-4{\dot{x}}_{0}^{2} \end{array}\right)c^{4}\\ +\left(2\alpha \lambda +4\alpha +2\lambda \rho^{2}+4\rho^{2}-3\beta \lambda x_{0}^{2}-\beta x_{0}^{2}\right)c^{6}+\beta c^{8}=0. \end{array}$$

### 2.7. Third Case: Quadratically Damped and Forced Oscillator

Let us consider the i.v.p.

(71) $$\begin{array}{c} \ddot{x}+\varepsilon \dot{x}\left|\dot{x}\right|+\alpha x+\beta x^{3}+\gamma x^{5}=F_{0}\cos\left(\omega t\right)+F_{1}\sin\left(\omega t\right),x\left(0\right)=x_{0}\text{ and }x^{\prime }\left(0\right)={\dot{x}}_{0}. \end{array}$$

Assume the ansatz

(72) $$\begin{array}{c} x\left(t\right)=u\left(t\right)+d_{0}\cos\left(\omega t\right)+d_{1}\sin\left(\omega t\right), \end{array}$$

where the function *u*=*u*(*t*) is a solution to some i.v.p.

(73) $$\begin{array}{c} \ddot{u}+\varepsilon \dot{u}\left|\dot{u}\right|+pu+qu^{3}+\gamma u^{5}=0,u\left(0\right)=x_{0}-d_{0}\text{ and }u^{\prime }\left(0\right)={\dot{x}}_{0}-d_{1}\omega . \end{array}$$

The suitable constants *p*, *q*, *r*, *d*_0_, and *d*_1_ are to be determined. Define the residual function

(74) $$\begin{array}{c} R\left(t\right)=\ddot{x}\left(t\right)+\varepsilon \dot{x}\left(t\right)\left|\dot{x}\left(t\right)\right|+\alpha x\left(t\right)+\beta x^{3}\left(t\right)+\gamma x^{5}\left(t\right)-F_{0}\cos\left(\omega t\right)-F_{1}\sin\left(\omega t\right). \end{array}$$

The expression *R*(*t*) contains many terms. Equating some coefficients of *v*(*t*), *v*′(*t*), sin(*jωt*), and cos(*jωt*) (*j*=1,2,3,…), we obtain the following algebraic system:

(75) $$\begin{array}{c} 8\alpha d_{1}+6\beta d_{1}^{3}+6\beta d_{0}^{2}d_{1}+5\gamma d_{1}^{5}+10\gamma d_{0}^{2}d_{1}^{3}+5\gamma d_{0}^{4}d_{1}-8d_{1}\omega^{2}-8F_{1}=0, \end{array}$$

(76) $$\begin{array}{c} 8\alpha d_{0}+6\beta d_{0}^{3}+6\beta d_{1}^{2}d_{0}+5\gamma d_{0}^{5}+10\gamma d_{1}^{2}d_{0}^{3}+5\gamma d_{1}^{4}d_{0}-8d_{0}\omega^{2}-8F_{0}=0, \end{array}$$

(77) $$\begin{array}{c} 8\alpha +12\beta d_{0}^{2}+12\beta d_{1}^{2}+15\gamma d_{0}^{4}+30\gamma d_{1}^{2}d_{0}^{2}+15\gamma d_{1}^{4}-8p=0, \end{array}$$

(78) $$\begin{array}{c} \beta +5\gamma d_{0}^{2}+5\gamma d_{1}^{2}-q=0, \end{array}$$

(79) $$\begin{array}{c} \gamma -r=0. \end{array}$$

From equations (77)–(79), we obtain

(80) $$\begin{array}{c} .p=\alpha +\frac{3}{8}\left(d_{0}^{2}+d_{1}^{2}\right)\left(4\beta +5\gamma \left(d_{0}^{2}+d_{1}^{2}\right)\right),q=\beta +5\gamma \left(d_{0}^{2}+d_{1}^{2}\right),r=\gamma . \end{array}$$

Decupling equations (75) and (76) by means of their eliminants gives

(81) $$\begin{array}{c} \left(5\gamma F_{0}^{4}+10\gamma F_{1}^{2}F_{0}^{2}+5\gamma F_{1}^{4}\right)d_{0}^{5}+\left(6\beta F_{0}^{4}+6\beta F_{1}^{2}F_{0}^{2}\right)d_{0}^{3}+\left(8\alpha F_{0}^{4}-8F_{0}^{4}\omega^{2}\right)d_{0}-8F_{0}^{5}=0, \end{array}$$

(82) $$\begin{array}{c} \left(5\gamma F_{0}^{4}+10\gamma F_{1}^{2}F_{0}^{2}+5\gamma F_{1}^{4}\right)d_{1}^{5}+\left(6\beta F_{1}^{4}+6\beta F_{0}^{2}F_{1}^{2}\right)d_{1}^{3}+\left(8\alpha F_{1}^{4}-8F_{1}^{4}\omega^{2}\right)d_{1}-8F_{1}^{5}=0. \end{array}$$

We choose the least in magnitude real root *d*_0_  to quintic (81) and the least in magnitude real root *d*_1_  to quintic (82). Assuming that the forces *F*_0_ and *F*_1_ are small in magnitude, we have the following approximations for these roots:

(83) $$\begin{array}{c} d_{0}=\frac{4F_{0}{\left(\alpha -\omega^{2}\right)}^{2}}{4{\left(\alpha -\omega^{2}\right)}^{3}+3\beta \left(F_{0}^{2}+F_{1}^{2}\right)},\\ d_{1}=\frac{4F_{1}{\left(\alpha -\omega^{2}\right)}^{2}}{4{\left(\alpha -\omega^{2}\right)}^{3}+3\beta \left(F_{0}^{2}+F_{1}^{2}\right)}. \end{array}$$

More precise approximations are

(84) $$\begin{array}{c} d_{0}=\frac{2F_{0}\sqrt{\alpha -\omega^{2}}}{{\left(\alpha -\omega^{2}\right)}^{3/2}\pm \sqrt{{\left(\alpha -\omega^{2}\right)}^{3}+3\beta \left(F_{0}^{2}+F_{1}^{2}\right)}},\\ d_{1}=\frac{2F_{1}\sqrt{\alpha -\omega^{2}}}{{\left(\alpha -\omega^{2}\right)}^{3/2}\pm \sqrt{{\left(\alpha -\omega^{2}\right)}^{3}+3\beta \left(F_{0}^{2}+F_{1}^{2}\right)}}. \end{array}$$

## 3. Further Applications

Suppose we are given a quadratically damped oscillator

(85) $$\begin{array}{c} \ddot{x}+\varepsilon \dot{x}\left|\dot{x}\right|+h\left(x\right)=0,x\left(0\right)=x_{0}\text{ and }x^{\prime }\left(0\right)={\dot{x}}_{0}, \end{array}$$

where *h*(−*x*)=−*h*(*x*) is an odd continuous function. Suppose that |*x*| ≤ *M*. Then, we may approximate the function *h*(*x*) by means of the following quintic polynomial:

(86) $$\begin{array}{c} h\left(x\right)\approx \alpha x+\beta x^{3}+\gamma x^{5}, \end{array}$$

where

(87) $$\begin{array}{c} \alpha =\frac{h\left(-\left(M/\sqrt{2}\right)\right)-h\left(M/\sqrt{2}\right)-\left(5+3\sqrt{3}\right)h\left(-\left(1/2\right)\sqrt{2-\sqrt{3}}M\right)+\left(5+3\sqrt{3}\right)h\left(\left(1/2\right)\sqrt{2-\sqrt{3}}M\right)-\left(3\sqrt{3}-5\right)\left(h\left(-\left(1/2\right)\sqrt{2+\sqrt{3}}M\right)-h\left(\left(1/2\right)\sqrt{2+\sqrt{3}}M\right)\right)}{3\sqrt{2}M}, \end{array}$$

(88) $$\begin{array}{c} \beta =\frac{\sqrt{2}\left(-8h\left(-\left(M/\sqrt{2}\right)\right)+8h\left(\left(M/\sqrt{2}\right)\right)+\left(7+5\sqrt{3}\right)h\left(-\left(1/2\right)\sqrt{2-\sqrt{3}}M\right)-\left(7+5\sqrt{3}\right)h\left(\left(1/2\right)\sqrt{2-\sqrt{3}}M\right)+\left(5\sqrt{3}-7\right)\left(h\left(-\left(1/2\right)\sqrt{2+\sqrt{3}}M\right)-h\left(\left(1/2\right)\sqrt{2+\sqrt{3}}M\right)\right)\right)}{3M^{3}}, \end{array}$$

(89) $$\begin{array}{c} \gamma =\frac{4\sqrt{2}\left(2h\left(-\left(M/\sqrt{2}\right)\right)-2h\left(\left(M/\sqrt{2}\right)\right)-\left(1+\sqrt{3}\right)h\left(-\left(1/2\right)\sqrt{2-\sqrt{3}}M\right)+\left(1+\sqrt{3}\right)h\left(\left(1/2\right)\sqrt{2-\sqrt{3}}M\right)-\left(\sqrt{3}-1\right)\left(h\left(-\left(1/2\right)\sqrt{2+\sqrt{3}}M\right)-h\left(\left(1/2\right)\sqrt{2+\sqrt{3}}M\right)\right)\right)}{3M^{5}}. \end{array}$$

Then, the i.v.p. (85) is reduced to the i.v.p. (1).

**Example 4 .**

Consider the motion of a satellite along a path that is equidistant from two identical massive stars with mutually interacting gravitational fields. If the distance between the two stars is 2d and the coordinate of the satellite motion is *x*, then the equation of motion of the satellite is given as

(90) $$\begin{array}{c} \ddot{x}+\varepsilon \left|\dot{x}\right|\dot{x}+\frac{2mx}{{\left(d^{2}+x^{2}\right)}^{3/2}}=0,x\left(0\right)=x_{0}\text{ and }x^{\prime }\left(0\right)={\dot{x}}_{0}, \end{array}$$

where *m*  is the mass of a star and the restoring force is

(91) $$\begin{array}{c} h\left(x\right)=\frac{2mx}{{\left(d^{2}+x^{2}\right)}^{3/2}}. \end{array}$$

The nonlinear restoring force is an irrational force because of the bottom square root. The restoring force spikes near the origin. The spikes indicate the point when the satellite is most influenced by the mutual gravitational field of the stars. Away from the origin, the restoring force decreases gradually and approaches the horizontal axis asymptotically. This means that the satellite is far away from the stars and experiences a much smaller gravitational force.

## 4. Conclusions

We have obtained approximate analytical solutions to the quadratically damped Duffing oscillator equation by means of an elementary approach. We introduced a parameter technique that allowed us to optimize the obtained solution. The results are also valid for the linear quadratically damped oscillator $\ddot{x}+\varepsilon \dot{x}\left|\dot{x}\right|+\alpha x=0$. Also, a more general quadratically damped oscillator $\ddot{x}+\varepsilon \dot{x}\left|\dot{x}\right|+h\left(x\right)=0$ may be solved for any odd parity function *h*(*x*). We also show the way to solve quadratically damped forced oscillators having the form $\ddot{x}+\varepsilon \dot{x}\left|\dot{x}\right|+h\left(x\right)=F\left(t\right)$ for any continuous functions *h*(*x*) and *F*(*t*) with *h*(−*x*)=−*h*(*x*).

The quadratically damped cubic-quintic oscillator having both forcing term and quadratic damping term has been analyzed analytically using some highly accurate approaches. The proposed analytical techniques may be applied to solve other strongly nonlinear oscillators.

## Figures and Tables

**Figure 1:**
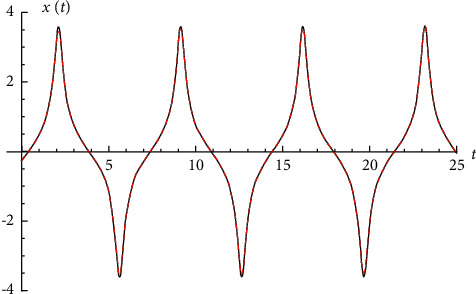
Comparison between the exact solution and the Runge–Kutta numerical solution for i.v.p. (24).

## Data Availability

No data were used to support this study.
